# Early readmission after adrenalectomy for pheochromocytoma. A retrospective study

**DOI:** 10.1007/s00423-025-03719-3

**Published:** 2025-05-08

**Authors:** Tamer A. A. M. Habeeb, Abd Al-Kareem Elias, Abdelmonem A. M. Adam, Mohamed A. Gadallah, Saad Mohamed Ali Ahmed, Ahmed Khyrallh, Mohammed H. Alsayed, Esmail Tharwat Kamel Awad, Emad A. Ibrahim, Mohamed Fathy Labib, Sobhy Rezk Ahmed Teama, Mahmoud Hassib Morsi Badawy, Mohamed Ibrahim Abo Alsaad, Abouelatta KH Ali, Hamdi Elbelkasi, Mahmoud Ali Abou Zaid, Ibtsam AbdElMaksoud Mohamed El Shamy, Boshra Ali Ali El-houseiny, Mahmoud El Azawy, Ahmed Elhoofy, Ali Hussein Khedr, Abdelrahman Mohamed Hasanin Nawar, Ahmed Salah Arafa, Ahmed Mesbah Abdelaziz, Abdelfatah H. Abdelwanis, Mostafa M. Khairy, Ahmed M. Yehia, Ahmed Kamal el Taher

**Affiliations:** 1 Department of General Surgery, Faculty of Medicine, Zagazig University, Zagazig, Egypt; 2 Department of General Surgery, Faculty of Medicine, Al-Azhar University, Assuit Branch, Assuit, Egypt; 3 General Surgery Department, Faculty of Medicine, Al-Azhar University, Cairo, Egypt; 4 General Surgery-Faculty of Medicine.Merit University, Sohag, Egypt; 5 Misr University for Science and Technology, Cairo, Egypt; 6 Mataryia Teaching Hospital (GOTHI), Cairo, Egypt; 7 General Surgery Department, El Mahala Hepatic Institute, Al Gharbia, Egypt; 8 Department of General Surgery, Faculty of Medicine for Girls, Al Azhar University, Cairo, Egypt; 9 Surgery Department, Faculty of Medicine, Helwan University, Helwan, Egypt; 10 General Surgery Ain Shams University, Cairo, Egypt; 11 Department of Surgery National Hepatology and Tropical Medicine Research Institute (NHTMRI), Cairo, Egypt

**Keywords:** Laparoscopic adrenalectomy, Open adrenalectomy, Postoperative complications, Pheochromocytoma

## Abstract

**Purpose:**

Adrenalectomy for pheochromocytoma (PHEO) presents a significant challenge due to the high incidence of early hospital readmission (ER). This study evaluated the incidence and risk factors of ER for PHEO within 30 days of adrenalectomy.

**Methods:**

A retrospective analysis of 346 patients > 18 years with unilateral PHEO who underwent adrenalectomy between September 2012 and September 2024. The patients were categorised into ER (*n* = 49) and no ER (*n* = 297) groups. Logistic regression analyses were performed to predict risk factors for ER.

**Results:**

The most common causes of ER were postoperative maintained hypotension (42.9%), bleeding (6.1%), ileus (24.5%), wound infection (4.1%), hyperkalemia (8.2%), pneumonia (2%), intra-abdominal abscess (2%), acute MI (4.1%), and colonic injury (6.1%). Most postoperative complications were Clavien-Dindo grade II (*n* = 40, 81.6%). Two perioperative deaths (4%) occurred in the ER group. Logistic regression showed that low body mass index (OR 0.849, 95% CI, 0.748–0.964; *p* = 0.012), tumor size < 5 cm (OR 0.096, 95% CI, 0.030–0.310; *p* < 0.001), and low ASA (OR 0.435, 95% CI, 0.249–0.761; *p* = 0.003) were associated with risk reduction for ER while malignancy (OR 5.302, 95% CI, 1.214–23.164; *p* = 0.027), open approach(OR 12.247, 95% CI, 5.227–28.694; *p* < 0.001), and intraoperative complications (OR 19.149, 95% CI, 7.091–51.710; *p* < 0.001) were associated with risk increase of ER.

**Conclusion:**

Postoperatively maintained hypotension and ileus were the most common causes of ER. Low body mass index, tumour size < 5 cm, and low ASA were risk reductions for ER, while malignancy, open approach, and intraoperative complications were the independent risk increase factors.

## Introduction

Pheochromocytoma (PHEO) originates from adrenomedullary cells that secrete adrenaline and noradrenaline [1]. The Clinical symptoms of tumour catecholamine overproduction range from silent to sudden death [2, 3]. Surgery for PHEO may involve either an open or laparoscopic adrenalectomy. Emerging technologies now encompass minimally invasive laparoscopic adrenalectomy (LA), which offers the benefits of excellent surgical view, precise dissection, and less tumour manipulation. It is safe feasible, and is associated with lower morbidity [4]. Gagner et al. [5] pioneered transperitoneal LA. Subsequently, numerous surgeons have advocated transperitoneal LA because of its recognisable anatomy and wide operational field [6].

Adrenal glands exhibit various anatomical relationships. The right adrenal gland lies partially behind the inferior vena cava (IVC), close to the liver and duodenum, and drains through the small right adrenal vein directly into the IVC posterolaterally. The left adrenal gland is intimately associated with the colon, pancreatic tail, and spleen and drains through the longer left adrenal vein into the left renal vein. Technical challenges can complicate adrenalectomy, thereby increasing the risk of complications [7–9]. The occurrence of postoperative complications following PHEO surgery varies between 11.4% and 29.8%, with comorbidities, tumour size, catecholamine levels, and surgical techniques correlating with an increased risk of such complications [3, 10, 11].

Various early hospital readmission (ER) risk factors have been addressed [12, 13]. Preoperative assessment of such risk factors is crucial for surgeons to determine the likelihood of ER, formulate a surgical strategy to minimise readmission rates and enhance perioperative surgical outcomes. Understanding these risk factors enables us to inform patients and include them in the decision-making process regarding surgical treatment. We have previously assessed the risk variables for intraoperative hemodynamic instability [14]. Recognising the risk factors for ER should enhance the perioperative treatment. This study assessed ER incidence and risk predictors within 30 days of PHEO surgery owing to complications.

## Material and methods

The University Research Ethics Board approved the study protocol (IRB number: 10281212025) and was registered at www.clinicaltrials.gov (NCT06697652). This study adhered to the STROCSS Guidelines [15]. The study team did not plan prior protocols.

**Fig. 1:**
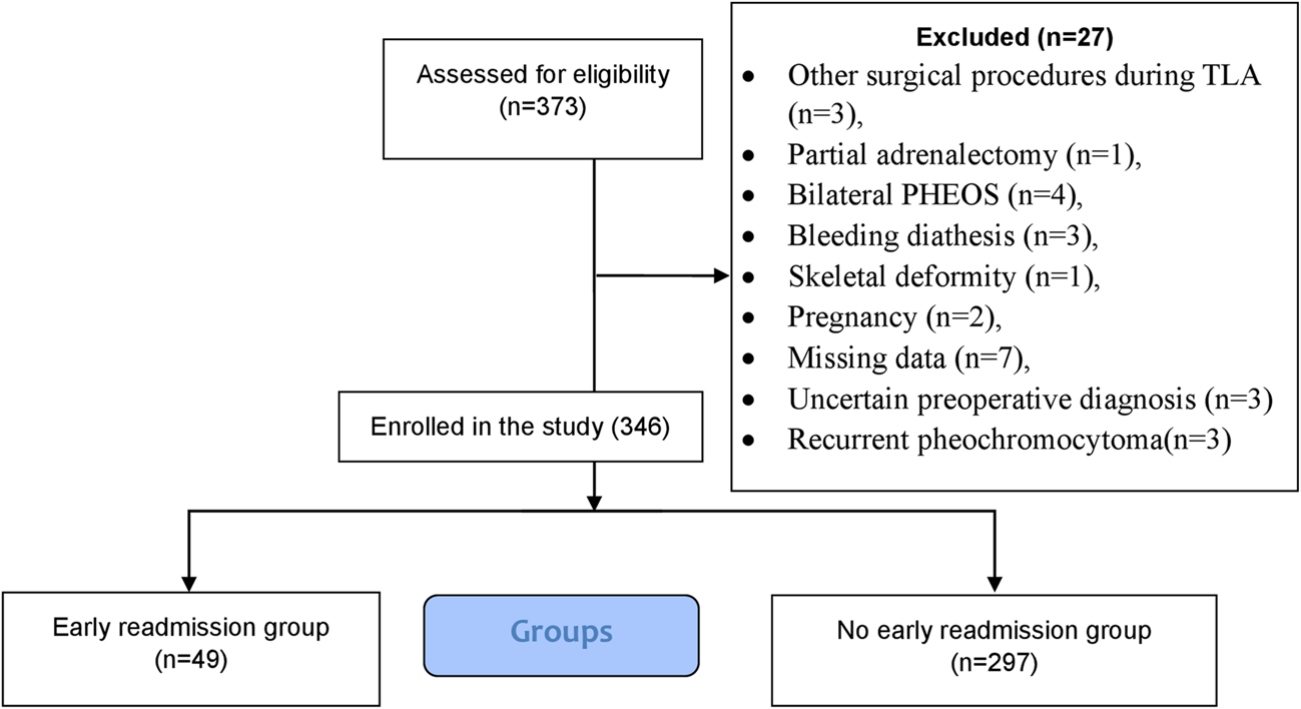
Flow Diagram of inclusion and exclusion criteria of studied patients

Study design and eligibility criteria: 346 consecutive patients > 18 years with unilateral PHEO (benign or malignant) who underwent open adrenalectomy or transperitoneal LA between September 2012 and September 2024 were retrospectively analysed. The patients were divided into ER (*n* = 49) and no ER (*n* = 297) groups. The diagnosis was confirmed biochemically, radiologically [16], and by postoperative pathological examination. Figure 1 illustrates a flowchart detailing the inclusion and exclusion criteria for the study participants.

Definition of Outcomes (endpoints) and measurements: The outcomes were the incidence and predictors of ER within 30 days after adrenalectomy for PHEO. Following adrenergic receptor blockade during the pre-induction phase, blood pressure was < 130/80 mmHg, with a heart rate of < 80 beats per minute in the supine position and < 100 beats per minute in the standing position [17]. Tumor sizes were documented based on preoperative CT findings, with tumours measuring ≥ 5 cm classified as"large"[18]. Intraoperative hemodynamic instability (HDI) is characterised by systolic blood pressure (SBP) > 200 mmHg for > one minute or a mean arterial pressure (MAP) < 60 mmHg, necessitating the administration of intravenous vasopressors (norepinephrine or epinephrine) or vasodilators (nitroprusside) to sustain normal blood pressure during the procedure [19]. Maintained hypotension is defined as the requirement for continuous saline and vasopressor infusion (norepinephrine or epinephrine) to sustain a systolic blood pressure (SBP) of at least 90 mm Hg for more than 24 h postoperatively [20]. Operative time was defined as the duration (in minutes) from skin or port-site incision to skin closure. The American Society of Anesthesiologists (ASA) classification was rated from I to IV [21]. The Clavien-Dindo classification was employed to assess morbidity [22]. Genetic analyses were not performed in this study.

### Perioperative approaches

All patients followed a standard operating procedure (SOPS) to the guidelines [23]. All patients with PHEO received preoperative doxazosin, bunazosin [24], phenoxybenzamine [25], and atenolol (beta-blockers) [26]. As required, hypotension was controlled during and after surgery using plasma expanders or norepinephrine infusion. Open adrenalectomy [27] and transperitoneal laparoscopic adrenalectomy [28] were conducted as previously outlined. All procedures were conducted by proficient endocrine surgeons, each performing over 30 laparoscopic adrenalectomies, the requisite minimum to surmount the learning curve [29]. We conducted clipless laparoscopic adrenalectomy by occluding the adrenal arteries and veins using a harmonic scalpel (Johnson and Johnson) or LigaSure (Covidien-Medtronic) [30]. When clips were required, we used titanium clips or Hemo-lok clips (Weck Closure Systems, Research Triangle Park, NC, USA). All specimens were obtained using an endoscopic pouch (U.S. Surgical, Norwalk, CT, USA) and were sent for histological analysis. Implementation of drainage is contingent on the selection of the primary surgeon. Postoperatively, all patients were admitted to the ICU and offered typical enhanced recovery protocols, including early movement and nutrition. Blood pressure was monitored in the ICU for the first 48 h postoperatively, followed by regular intervals (every 4 h) until discharge. Patients requiring vasopressors beyond 24 h were indicated for readmission.

### Statistical methods

Statistical analyses were conducted using SPSS 28 (IBM Corp., Armonk, New York, USA), employing the Kolmogorov–Smirnov test for data visualisation and quantitative analysis for normality assessment. We employed the independent t-test or Mann–Whitney U-test to assess the distribution of normally and non-normally distributed quantitative variables across groups. We used the chi-square or Fisher's exact test to compare categorical data. Univariate and multivariate logistic regression analyses were used to predict early readmission and calculate odds ratios (OR) with 95% confidence intervals (CI). Only variables with a *p*-value < 0.25 in the univariate analysis were included in the multivariable analysis. All statistical tests were two-tailed, with significance set at *p* < 0.05.

## Results

Three hundred forty-six patients (*n* = 346) were categorised into ER (*n* = 49) and no ER (*n* = 297) groups. Table 1 shows the baseline patient and tumour data. We found no significant differences between the two groups regarding the preoperative data, except for smoking(47% vs 23.6%,*p* < 0.001), Body Mass Index (BMI) (33.7 ± 3.2 vs 31.4 ± 3.8, *P* < 0.001), family history(71.4% vs 16.5%, *p* = 0.042), PASS score(*p* < 0.001), pathological type of the tumour (benign or malignant) (*p* = 0.049), mean tumour size(6.5 ± 1.8 vs 5.07 ± 1.3, *p* = < 0.001), tumour diameter > 5 cm(91.8% vs 50.5%, *p* < 0.001) ASA(*p* < 0.001), median preoperative diastolic blood pressure before alpha-blocker use [96(92–100) vs. 92(89–98), *p* = 0.007], median preoperative SBP after alpha-blocker use [125(119–127) vs. (120–127), *p* = 0.026), alpha-blocker usage(*p* < 0.001), median 24 h urinary metanephrine, nor metanephrine [3.8(3.25–3.9) vs 3.1(2.8–3.9), *p* = 0.006], and previous upper abdominal surgery(46.9% vs 12.1%, *p* = 0.001) for ER and no ER groups, respectively. The median patient age was 48(45–53) and 46(41.5–53) years (*p* = 0.22), and 8(16.3%) vs. 26(9.8%) were retrocaval PHEO (*p* = 0.09). The most common comorbidities were DM (28.6% vs 28.3%) and hypertension (10.2% vs 9.4%) (*p* = 0.690) in both groups respectively.

**Table 1:** Patients and tumor characteristics in the studied groups

		Early readmission (*n* = 49) (%)	No early readmission (*n* = 297) (%)	*p*-value
Age(median, IQR)		48(45–53)	46(41.5–53)	0.222
Sex	Male	34(69.4%)	186(62.6%)	0.362
	Female	15(30.6%)	111(37.4%)	
Smoker	Smoker	23(47%)	70(23.6%)	< 0.001*
	Non-smoker	26(53.1%)	227(76.4%)	
BMI (mean ± SD)		33.7 ± 3.2	31.4 ± 3.8	< 0.001*
Family history of PHEO		35(71.4%)	49(16.5%)	0.042*
Side of PHEO	Right-sided	17(34.7%)	132(44.4%)	0.202
	Left-sided	32(65.3%)	165(55.6%)	
PASS score	< 4	6(12.2%)	266(89.6%)	< 0.001*
	≥ 4	43(87.8%)	31(10.4%)	
Benign or malignant	Benign PHEO	9(18.4%)	270(90.9%)	0.049*
	Malignant PHEO	40(81.6%)	27(9.1%)	
Tumor size (cm)(mean SD)		6.5 ± 1.8	5.07 ± 1.3	< 0.001*
Tumor size	< 5 cm	4(8.2%)	147(49.5%)	< 0.001*
	> 5 cm	45(91.8%)	150(50.5%)	
Retrocaval PHEO	no	41(83.7%)	271(91.2%)	0.09
	yes	8(16.3%)	26(9.8%)	
ASA	II	6(12.2%)	174(58.6%)	< 0.001*
	III	39(79.6%)	89(30%)	
	IV	4(8.2%)	34(11.4%)	
Comorbidities	No comorbidities	30(61.2%)	172(57.9%)	0.690
	DM	14(28.6%)	84(28.3%)	
	HTN	5(10.2%)	28(9.4%)	
	Previous MI	0(0.00%)	6(2%)	
	Previous stroke	0(0.00%)	7(2.4%)	
	CHD	0(0.00%)	0(0.00%)	
Preoperative predominant clinical symptoms	HTN	31(63.3%)	170(57.2%)	0.157
	Sweating	6(12.2%)	52(17.5%)	
	Palpitation	3(6.1%)	43(14.5%)	
	Headache	9(18.4%)	32(10.8%)	
Preoperative SBP (before alpha-blocker)(median, IQR)		147(140–150)	145(142–150)	0.438
Preoperative DBP (before alpha-blocker))(median, IQR)		96(92–100)	92(89–98)	0.007*
Preoperative SBP after alpha blocker(pre-induction) (median, IQR)		125(119–127)	125(120–127)	0.026*
Preoperative DBP after alpha blocker(pre-induction) (median, IQR)		77(71–78)	77(69–79)	0.904
Alpha blocker	Bunazocin	1(2%)	31(10.4%)	< 0.001*
	Doxazocin	29(59.2%)	114(38.4%)	
	Phenoxypenzamine	19(38.8%)	152(51.2%)	
Beta-blocker		9(18.4%)	43(14.5%)	0.480
24 h urinary epinephrine (microgram/24) (*n* = 0–20) (median, IQR)		89(74–100)	87(73–95)	0.333
24 h urinary nor epinephrine (microgram/24)(*n* = 15–80)(median, IQR)		134(125–136)	133(125 −134)	0.271
24 h urinary metanephrine and normetanephrine(mg/24)(*n* = 0–1.2 mg/day)(median, IQR)		3.8(3.25–3.9)	3.1(2.8–3.9)	0.006*
24 h urinary VMA (*n* = 0–7.9 mg/day) (median, IQR)		34(23.5–41)	35(24–41)	0.490
Plasma epinephrine(pg/ml)(*n* = 4–83 pg/ml)		126(121–145)	131(122–152)	0.887
Plasma norepinephrine(pg/ml)(*n* = 80–498 pg/ml)(median, IQR)		697(658–837)	735(640–831.5)	0.955
Previous upper abdominal surgery		23(46.9%)	36(12.1%)	0.001*

*IQR* Interquartile range, *PHEO* Pheochromocytoma, *PASS* Pheochromocytoma of the Adrenal Gland Scaled Score, *SBP* systolic blood pressure, *DBP* diastolic blood pressure, *ASA* American Society of Anesthesiologist, *CHD* coronary heart disease, *MI* myocardial infarction, *DM* diabetes mellitus, *BMI* body mass index, *HTN* hypertension. *statistically significant

Table 2 shows intraoperative data. No statistically significant difference between the groups as regards intraoperative data except that the ER group had longer median operative time (158(136–198.5) vs 134(122–145) min, *p* < 0.001), more intraoperative complications (41 (83.7%) vs 1(0.3%) (*p* < 0.001) and higher conversion(16(32.7%) vs 11(3.7%) (*p* < 0.001). In this study, the most common intraoperative complication was bleeding from adrenal vein injuries in both groups. Moreover, the commonest causes of conversion were uncontrolled bleeding from the adrenal vein in the early readmission group and adhesion with difficult dissection in the no early readmission group.

**Table 2:** Intraoperative data of the studied groups

		Early readmission (*n* = 49) (%)	No early readmission (*n* = 297) (%)	*P* value
Approach(open or laparoscope)	Open	24(49%)	29(9.8%)	< 0.001*
	Laparoscope	25(51%)	268(90.2%)	
Operative time(median,IQR)		158 (136–198.5)	134(122–145)	< 0.001*
Blood loss(ml) (median,IQR)		230(194.5–274)	210(187–269)	0.163
Intraoperative hemodynamic instability		10(20.4%)	36(12.1%)	0.113
Forms of intraoperative hemodynamic instability	Hypertensive crisis	8(16.3%)	32(10.8%)	0.195
	Tachycardia(> 100 bpm)	2(4.1%)	4(1.3%)	
Intraoperative complications	No intraoperative complications	8(16.3%)	296(99.7%)	< 0.001*
	Intraoperative bleeding from adrenal vein	20(40.8%)	1(0.3%)	
	Intraoperative acidosis	3(6.1%)	0	
	Intraoperative bleeding from IVC	10(20.4%)	0	
	Other causes of intraoperative bleeding	2(4.1%)	0	
	Liver injury	4(8.2%)	0	
	Splenic injury	1(2%)	0	
	Colonic injury	1(2%)	0	
Tumor rupture		1(2%)	13(4.4%)	0.442
Conversion		16(32.7%)	11(3.7%)	< 0.001*
Causes of conversion	Uncontrolled bleeding from adrenal vein	6(12.2%)	4(1.3%)	< 0.001*
	Adhesion with difficult dissection	2(4.1%)	7(2.4%)	
	Uncontrolled bleeding from IVC	1(2%)	0	
	Intraoperative recurrent hemodynamic instability	3(6.1%)	0	
	Uncontrolled bleeding from splenic injury	3(6.1%)	0	
	Left colonic injury	1(2%)	0	

*IQR* Interquartile range, *IVC* Inferior vena cava. *statistically significant

Table 3 shows postoperative data. The commonest causes of early postoperative complications and ER were postoperative maintained hypotension 21(42.9%), bleeding 3(6.1%), ileus 12(24.5%), wound infection 2(4.1%), hyperkalemia4 (8.2%), pneumonia1 (2%), intra-abdominal abscess1 (2%), acute MI 2(4.1%), and colonic injury3(6.1%). The ER group is associated with a statistically significant higher median hospital stay [4(3–8.5) vs. 4(3–4), *p* < 0.001] and CD classification (*p* < 0.001). Most postoperative complications were Clavien-Dindo grade II (*N* = 40(81.6%), *p* < 0.001). There is no statistically significant difference between both groups regarding postoperative 30-day mortality (*p* = 0.339). There were two perioperative deaths (4%) ten days after surgery due to acute respiratory failure and myocardial infarction. Reoperation occurred in three patients who were diagnosed with postoperative colonic injury.

**Table 3:** Postoperative data of the studied groups

		Early readmission (*n* = 49)(%)	No early readmission (*n* = 297)(%)	*P* value
Hospital stay(median, IQR)		4(3–8.5)	4(3–4)	< 0.001*
Early postoperative complications	Maintained hypotension	21(42.9%)	0(0.00%)	< 0.001*
	Bleeding	3(6.1%)	0(0.00%)	
	Ileus	12(24.5%)	0(0.00%)	
	Wound infection	2(4.1%)	0(0.00%)	
	Hyperkalemia	4(8.2%)	0(0.00%)	
	Pneumonia	1(2%)	0(0.00%)	
	Intra-abdominal abscess	1(2%)	0(0.00%)	
	Acute MI	2(4.1%)	0(0.00%)	
	Colonic injury	3(6.1%)	0(0.00%)	
Treatment of early readmission	Conservative treatment + k losing diuretics	4(8.2%)	0(0.00%)	< 0.001*
	Conservative treatment antibiotic	2(4.1%)	0(0.00%)	
	Conservative treatment blood transfusion	3(6.1%)	0(0.00%)	
	Conservative treatment cardiac support	2(4.1%)	0(0.00%)	
	Conservative treatment + IV fluid Ryle	13(26.5%)	0(0.00%)	
	Conservative treatment respiratory support	1(2%)	0(0.00%)	
	Postoperative fluid and vasopressor	20(40.8%)	0(0.00%)	
	Radiological drainage	1(2%)	0(0.00%)	
	Surgical re-intervention	3(6.1%)	0(0.00%)	
Clavien-Dindo classification				< 0.001*
	Grade I	2(4.1%)	0(0.00%)	
	Grade II	40(81.6%)	0(0.00%)	
	Grade III	4(8.2%)	0(0.00%)	
	Grade IV	2(4.1%)	0(0.00%)	
Postoperative mortality(within 30 days)	No mortality	47(96%)	297(100%)	0.339
	Acute respiratory failure	1(2%)	0(0.00%)	
	Acute MI	1(2%)	0(0.00%)	

*IQR* Interquartile range, *MI* myocardial infarction. *statistically significant

Table 4 shows full details of postoperative complications.

**Table 4:** Details of causes of early readmission

Variable		Maintained hypotension (*n* = 21)	Bleeding (*n* = 3)	Ileus (*n* = 12)	Wound infection (*n* = 2)	Hyperkalemia (*n* = 4)	Pneumonia (*n* = 1)	Intra-abdominal abscess (*n* = 1)	Acute MI (*n* = 2)	colonic injury (*n* = 3)
Age(median)(years)		51	42	47	45	45	44	44	52	48
Sex	male	16(76.2%)	3(100%)	5(41.7%)	0(0.00%)	4(100%)	100%	0(0.00%)	2(100%)	3(100%)
	female	5(23.8%)	0(0.00%)	7(58.3%)	2(100%)	0(0.00%)	0(0.00%)	100%	0(0.00%)	0(0.00%)
Smoker	smoker	6(28.6%)	1(33.3%)	5(41.7%)	1(50%)	4(100%)	100%	0(0.00%)	2(100%)	3(100%)
	non smoker	15(71.4%)	2(66.7%)	7(58.3%)	1(50%)	0(0.00%)	0(0.00%)	100%	0(0.00%)	0(0.00%)
Body mass index		35	34	34	34	35	35	37	23	31
Family history of pheos		13(61.9%)	1(33.3%)	12(100%)	2(100%)	0(0.00%)	100%	100%	2(100%)	3(100%)
Side of pheo	right sided	8(38.1%)	2(66.7%)	1(8.3%)	1(50%)	4(100%)	100%	0(0.00%)	0(0.00%)	0(0.00%)
	left sided	13(61.9%)	1(33.3%)	11(91.7%)	1(50%)	0(0.00%)	0(0.00%)	100%	2(100%)	3(100%)
PASS score	≥ 4	16(76.2%)	3(100%)	12(100%)	2(100%)	4(100%)	100%	100%	2(100%)	2(66.7%)
	< 4	5(23.8%)	0(0.00%)	0(0.00%)	0(0.00%)	0(0.00%)	0(0.00%)	0(0.00%)	0(0.00%)	1(33.3%)
Benign or malignant	malignant pheo	12(57.1%)	3(100%)	12(100%)	2(100%)	4(100%)	100%	100%	2(100%)	3(100%)
	benign pheo	9(42.9%)	0(0.00%)	0(0.00%)	0(0.00%)	0(0.00%)	0(0.00%)	0(0.00%)	0(0.00%)	0(0.00%)
Tumor size (cm)		7.6	7.3	5.6	6.1	6.2	6.8	2.6	5.8	5.1
Tumor size	< 5 cm	0(0.00%)	0(0.00%)	3(25%)	0(0.00%)	0(0.00%)	0(0.00%)	100%	0(0.00%)	0(0.00%)
	> 5 cm	21(100%)	3(100%)	9(75%)	2(100%)	4(100%)	100%	0(0.00%)	2(100%)	3(100%)
Retrocaval pheochromocytoma	no	16(76.2%)	1(33.3%)	12(100%)	2(100%)	4(100%)	0(0.00%)	100%	2(100%)	3(100%)
	yes	5(23.8%)	2(66.7%)	0(0.00%)	0(0.00%)	0(0.00%)	100%	0(0.00%)	0(0.00%)	0(0.00%)
ASA	2	0(0.00%)	0(0.00%)	2(16.7%)	1(50%)	2(50%)	0(0.00%)	0(0.00%)	0(0.00%)	1(33.3%)
	3	19(90.5%)	3(100%)	8(66.7%)	1(50%)	2(50%)	100%	100%	2(100%)	2(66.7%)
	4	2(9.5%)	0(0.00%)	2(16.7%)	0(0.00%)	0(0.00%)	0(0.00%)	0(0.00%)	0(0.00%)	0(0.00%)
Comorbidities	no comorbidities	12(57.1%)	1(33.3%)	7(58.3%)	1(50%)	4(100%)	100%	100%	0(0.00%)	3(100%)
	DM	6(28.6%)	2(66.7%)	4(33.3%)	0(0.00%)	0(0.00%)	0(0.00%)	0(0.00%)	2(100%)	0(0.00%)
	hypertension	3(14.3%)	0(0.00%)	1(8.3%)	1(50%)	0(0.00%)	0(0.00%)	0(0.00%)	0(0.00%)	0(0.00%)
	previous myocardial infarction	0(0.00%)	0(0.00%)	0(0.00%)	0(0.00%)	0(0.00%)	0(0.00%)	0(0.00%)	0(0.00%)	0(0.00%)
	previous stroke	0(0.00%)	0(0.00%)	0(0.00%)	0(0.00%)	0(0.00%)	0(0.00%)	0(0.00%)	0(0.00%)	0(0.00%)
	coronary heart disease	0(0.00%)	0(0.00%)	0(0.00%)	0(0.00%)	0(0.00%)	0(0.00%)	0(0.00%)	0(0.00%)	0(0.00%)
Preoperative predominant clinical symptoms	hypertension	18(85.7%)	3(100%)	6(50%)	1(50%)	0(0.00%)	100%	0(0.00%)	2(100%)	0(0.00%)
	sweating	2(9.5%)	0(0.00%)	0(0.00%)	0(0.00%)	4(100%)	0(0.00%)	0(0.00%)	0(0.00%)	0(0.00%)
	palpitation	1(4.8%)	0(0.00%)	1(8.3%)	1(50%)	0(0.00%)	0(0.00%)	0(0.00%)	0(0.00%)	0(0.00%)
	headache	0(0.00%)	0(0.00%)	5(41.7%)	0(0.00%)	0(0.00%)	0(0.00%)	100%	0(0.00%)	3(100%)
preoperative SBP (before alpha blocker)		151	155	148	140	140	140	139	139	142
preoperative DBP (before alpha blocker)		97	107	96	96	92	94	88	88	100
preoperative SBP after alpha blocker(pre-induction)		123	124	121	120	110	128	125	128	125
preoperative DBP after alpha blocker(pre-induction)		75	79	76	69	71	77	71	70	73
Alpha blocker	Bunazocin	0(0.00%)	0(0.00%)	0(0.00%)	1(50%)	0(0.00%)	0(0.00%)	0(0.00%)	0(0.00%)	0(0.00%)
	Doxazocin	10(47.6%)	2(66.7%)	5(41.7%)	1(50%)	4(100%)	100%	100%	2(100%)	3(100%)
	Phenoxypenzamine	11(52.4%)	1(33.3%)	7(58.3%)	0(0.00%)	0(0.00%)	0(0.00%)	0(0.00%)	0(0.00%)	0(0.00%)
Beta blocker		6(28.6%)	1(33.3%)	2(16.7%)	0(0.00%)	0(0.00%)	0(0.00%)	0(0.00%)	0(0.00%)	0(0.00%)
24 h urinary epinephrine (microgram/24) (*n* = 0–20)		94	94	86	103	60	133	100	79	83
24 h urinary nor epinephrine(microgram/24)(*n* = 15–80)		128	137	134	134	133	134	133	134	125
24 h urinary metanephrine and nor metanephrine(mg/24)(*n* = 0–1.2 mg/day)		3.4	3.4	3.6	2.7	3.9	4.1	4.1	4.1	3.5
24 h urinary VMA (*n* = 0–7.9 mg/day)		32	34	38	38	34	24	36	21	24
Plasma epinephrine(pg/ml)(*n* = 4–83 pg/ml)		134	104	129	119	154	125	126	101	101
Plasma nor epinephrine(pg/ml)(*n* = 80–498 pg/ml)		778.52	714.67	804.25	687.00	536.00	689.00	589.00	658.00	687.00
Previous upper abdominal surgery	no	9(42.9%)	0(0.00%)	5(41.7%)	2(100%)	4(100%)	0(0.00%)	100%	2(100%)	3(100%)
	yes	12(57.1%)	3(100%)	7(58.3%)	0(0.00%)	0(0.00%)	100%	0(0.00%)	0(0.00%)	0(0.00%)
Approach (open or laparoscope)	open	7(33.3%)	1(33.3%)	8(66.7%)	2(100%)	1(25%)	0(0.00%)	100%	1(50%)	3(100%)
	laparoscope	14(66.7%)	2(66.7%)	4(33.3%)	0(0.00%)	3(75%)	100%	0(0.00%)	1(50%)	0(0.00%)
Operative time		184	188	149	149	121	214	148	122	135
Blood loss(ml)		231	200	224	192	311	199	184	245	184
Intraoperative hemodynamic instability	yes	5(23.8%)	2(66.7%)	2(16.7%)	1(50%)	0(0.00%)	0(0.00%)	0(0.00%)	0(0.00%)	0(0.00%)
	no	16(76.2%)	1(33.3%)	10(83.3%)	1(50%)	4(100%)	100%	100%	2(100%)	3(100%)
Forms of intraoperative hemodynamic instability	no	16(76.2%)	1(33.3%)	10(83.3%)	1(50%)	4(100%)	100%	100%	2(100%)	3(100%)
	hypertensive crisis	5(23.8%)	2(66.7%)	1(8.3%)	0(0.00%)	0(0.00%)	0(0.00%)	0(0.00%)	0(0.00%)	0(0.00%)
	tachycardia	0(0.00%)	0(0.00%)	1(8.3%)	1(50%)	0(0.00%)	0(0.00%)	0(0.00%)	0(0.00%)	0(0.00%)
Intraoperative complications	no intraoperative complications	0(0.00%)	0(0.00%)	3(25%)	0(0.00%)	0(0.00%)	0(0.00%)	0(0.00%)	2(100%)	3(100%)
	intraoperative bleeding from adrenal vein	20(95.2%)	0(0.00%)	0(0.00%)	0(0.00%)	0(0.00%)	0(0.00%)	0(0.00%)	0(0.00%)	0(0.00%)
	intraoperative acidosis	0(0.00%)	3(100%)	0(0.00%)	0(0.00%)	0(0.00%)	0(0.00%)	0(0.00%)	0(0.00%)	0(0.00%)
	intraoperative bleeding from IVC	1(4.8%)	0(0.00%)	9(75%)	0(0.00%)	0(0.00%)	0(0.00%)	0(0.00%)	0(0.00%)	0(0.00%)
	other causes of intraoperative bleeding	0(0.00%)	0(0.00%)	0(0.00%)	2(100%)	0(0.00%)	0(0.00%)	0(0.00%)	0(0.00%)	0(0.00%)
	liver injury	0(0.00%)	0(0.00%)	0(0.00%)	0(0.00%)	4(100%)	0(0.00%)	0(0.00%)	0(0.00%)	0(0.00%)
	splenic injury	0(0.00%)	0(0.00%)	0(0.00%)	0(0.00%)	0(0.00%)	100%	0(0.00%)	0(0.00%)	0(0.00%)
	colonic injury	0(0.00%)	0(0.00%)	0(0.00%)	0(0.00%)	0(0.00%)	0(0.00%)	100%	0(0.00%)	0(0.00%)
Tumor rupture	yes	1(4.8%)	0(0.00%)	0(0.00%)	0(0.00%)	0(0.00%)	0(0.00%)	0(0.00%)	0(0.00%)	0(0.00%)
	no	20(95.2%)	3(100%)	12(100%)	2(100%)	4(100%)	100%	100%	2(100%)	3(100%)
Conversion	yes	11(52.4%)	3(100%)	1(8.3%)	0(0.00%)	0(0.00%)	100%	0(0.00%)	0(0.00%)	0(0.00%)
	no	10(47.6%)	0(0.00%)	11(91.7%)	2(100%)	4(100%)	0(0.00%)	100%	2(100%)	3(100%)
Causes of conversion	uncontrolled bleeding from adrenal vein	2(9.5%)	3(100%)	1(8.3%)	0(0.00%)	0(0.00%)	0(0.00%)	0(0.00%)	0(0.00%)	0(0.00%)
	adhesion with difficult dissection	2(9.5%)	0(0.00%)	0(0.00%)	0(0.00%)	0(0.00%)	0(0.00%)	0(0.00%)	0(0.00%)	0(0.00%)
	uncontrolled bleeding from IVC	1(4.8%)	0(0.00%)	0(0.00%)	0(0.00%)	0(0.00%)	0(0.00%)	0(0.00%)	0(0.00%)	0(0.00%)
	intraoperative recurrent hemodynamic instability	2(9.5%)	0(0.00%)	0(0.00%)	0(0.00%)	0(0.00%)	100%	0(0.00%)	0(0.00%)	0(0.00%)
	uncontrolled bleeding from splenic injury	3(14.3%)	0(0.00%)	0(0.00%)	0(0.00%)	0(0.00%)	0(0.00%)	0(0.00%)	0(0.00%)	0(0.00%)
	left colonic injury	1(4.8%)	0(0.00%)	0(0.00%)	0(0.00%)	0(0.00%)	0(0.00%)	0(0.00%)	0(0.00%)	0(0.00%)
Hospital stay		3	1.5(1–2)	4.5(3–9)	6(3–9)	4(100%)	6(3–9)	3	9	7(3–12)
Treatment of early complications	conservative treatment + k losing diuretics	0(0.00%)	0(0.00%)	0(0.00%)	0(0.00%)	4(100%)	0(0.00%)	0(0.00%)	0(0.00%)	0(0.00%)
	conservative treatment + antibiotic	0(0.00%)	0(0.00%)	0(0.00%)	2(100%)	0(0.00%)	0(0.00%)	0(0.00%)	0(0.00%)	0(0.00%)
	conservative treatment + blood transfusion	0(0.00%)	3(100%)	0(0.00%)	0(0.00%)	0(0.00%)	0(0.00%)	0(0.00%)	0(0.00%)	0(0.00%)
	conservative treatment + cardiac support	0(0.00%)	0(0.00%)	0(0.00%)	0(0.00%)	0(0.00%)	0(0.00%)	0(0.00%)	2(100%)	0(0.00%)
	conservative treatment + IV fluid + Ryle	0(0.00%)	0(0.00%)	12(100%)	0(0.00%)	0(0.00%)	0(0.00%)	0(0.00%)	0(0.00%)	0(0.00%)
	conservative treatment + respiratory support	0(0.00%)	0(0.00%)	0(0.00%)	0(0.00%)	0(0.00%)	100%	0(0.00%)	0(0.00%)	0(0.00%)
	postoperative fluid and vasopressor	21(100%)	0(0.00%)	0(0.00%)	0(0.00%)	0(0.00%)	0(0.00%)	0(0.00%)	0(0.00%)	0(0.00%)
	radiological drainage	0(0.00%)	0(0.00%)	0(0.00%)	0(0.00%)	0(0.00%)	0(0.00%)	100%	0(0.00%)	0(0.00%)
	surgical re-intervention	0(0.00%)	0(0.00%)	0(0.00%)	0(0.00%)	0(0.00%)	0(0.00%)	0(0.00%)	0(0.00%)	3(100%)
Clavien—Dindo classification	Grade 0	0(0.00%)	0(0.00%)	0(0.00%)	0(0.00%)	0(0.00%)	0(0.00%)	0(0.00%)	0(0.00%)	0(0.00%)
	Grade I	0(0.00%)	0(0.00%)	0(0.00%)	2(100%)	0(0.00%)	0(0.00%)	0(0.00%)	0(0.00%)	0(0.00%)
	Grade II	21(100%)	3(100%)	12(100%)	0(0.00%)	4(100%)	0(0.00%)	0(0.00%)	0(0.00%)	0(0.00%)
	Grade III	0(0.00%)	0(0.00%)	0(0.00%)	0(0.00%)	0(0.00%)	0(0.00%)	100%	0(0.00%)	3(100%)
	Grade IV	0(0.00%)	0(0.00%)	0(0.00%)	0(0.00%)	0(0.00%)	100%	0(0.00%)	2(100%)	0(0.00%)
Postoperative mortality(30 days)	no mortality	21(100%)	3(100%)	10(83.3%)	2(100%)	4(100%)	100%	100%	2(100%)	3(100%)
	acute respiratory failure	0(0.00%)	0(0.00%)	1(8.3%)	0(0.00%)	0(0.00%)	0(0.00%)	0(0.00%)	0(0.00%)	0(0.00%)
	acute MI	0(0.00%)	0(0.00%)	1(8.3%)	0(0.00%)	0(0.00%)	0(0.00%)	0(0.00%)	0(0.00%)	0(0.00%)

*IQR* Interquartile range, *PHEO* Pheochromocytoma, *PASS* Pheochromocytoma of the Adrenal Gland Scaled Score, *SBP* systolic blood pressure, *DBP* diastolic blood pressure, *ASA* American Society of Anesthesiologist, *CHD* coronary heart disease, *MI* myocardial infarction, *DM* diabetes mellitus, *BMI* body mass index, *HTN* hypertension. *statistically significant

Table 5 shows the results of the logistic regression analysis for predicting ER. Multivariate Logistic regression analysis to predict ER showed that low body mass index (OR 0.849, 95% CI, 0.748–0.964; *p* = 0.012), tumour size < 5 cm (OR 0.096, 95% CI, 0.030–0.310; *p* < 0.001), and low ASA (OR 0.435, 95% CI, 0.249–0.761; *p* = 0.003) were associated with risk reduction for ER while malignancy (OR 5.302, 95% CI, 1.214–23.164; *p* = 0.027), open approach(OR 12.247, 95% CI, 5.227–28.694; *p* < 0.001), and intraoperative complications (OR 19.149, 95% CI, 7.091–51.710; *p* < 0.001) were associated with risk increase for ER.

**Table 5:** Univariate and multivariate logistic regression analysis to predict early readmission

	Univariate		Multivariate	
	OR (95% CI)	*P*-value	OR (95% CI)	*P*-value
Age	0.971(0.934–1.009)	0.30	-	
Sex	0.708(0.363–1.381)	0.311	-	
Low body mass index	0.850 (0.778- 0.929)	** < 0.001**	0.849 (0.748–0.964)	0.012*
PASS score	0.637 (0.262–1.548)	0.320	-	-
Malignant PHEO	2.838 (1.267–6.354)	**0.01**	5.302 (1.214–23.164)	0.027*
Tumour size < 5 cm	0.096 (0.034–0.275)	** < 0.001**	0.096 (0.030–0.310)	< 0.001*
Low ASA	0.449 (0.292–0.691)	** < 0.001**	0.435 (0.249–0.761)	0.003*
Preoperative SBP before alpha blocker	1.007 (0.977–1.038)	0.642	-	**-**
Open approach	8.593 (4.331—17.049)	** < 0.001**	12.247 (5.227–28.694)	< 0.001*
intraoperative HI	0.506 (0.232- 1.105)	< 0.001*	1.599 (0.559–4.572)	0.381
Intraoperative complications	0.089(0.038–0.207)	< 0.001*	19.149(7.091–51.710)	< 0.001*

*HI* Hemodynamic instability, *SBP* Systolic blood pressure, *PASS* Pheochromocytoma of the Adrenal Gland Scaled Score, *PHEO* Pheochromocytoma, *ASA* American Society of Anesthesiologist. *statistically significant

## Discussion

This study evaluated the incidence and risk factors of ER after open and laparoscopic adrenalectomies for PHEO. The most common causes of early postoperative complications and ER were postoperative maintained hypotension in 21(42.9%) and ileus in 12(24.5%). Multivariate Logistic regression analysis to predict ER showed that low body mass index, tumour size < 5 cm and low ASA were associated with risk reduction for ER whereas malignancy, open approach, and intraoperative complications were associated with an increased risk of ER.

The incidence of postoperative complications and ER in this analysis was 14.1%, comparable to that reported in other studies [3, 11, 31]. In this study, postoperative maintained hypotension was the most common cause of ER after surgery. It represented 21/49 (42.9%) of all cases requiring ER and 6% (21/346) of patients from the entire study group, which was significantly lower than other reports that reported frequencies of hypotension after adrenalectomy for PHEO of approximately 50% [20, 32–35]. The possible reasons for this variation were variations in sample size, the definition of maintained hypotension, patient selection, inclusion criteria, exclusion criteria, indications, variable preoperative medical preparation [17], anaesthesia, and variations in the approaches for adrenalectomy [19]. Ileus was the second most common cause of ER in our study and was presented in 12/49 patients (24.5%) with ER and in 12/346(3.5%) patients in the overall cohort. The causes of ileus may be open surgery and postoperative hypotension, which decrease the oxygen supply to the intestine [36, 37]. Lastly, improper use of catecholamine infusion during hypotension might have decreased splanchnic blood flow[38, 39]. Patients who underwent LA had earlier bowel recovery than those who underwent OA, similar to another study [40], and the open approach was a risk factor for ER in the current study. ER due to postoperative bleeding occurred in three patients (3/346, 0.9%), which required blood transfusion. Bleeding was uncommon in our series, contradicting previous reports [13, 31]. Although patients with PHEO are at a high risk of bleeding due to the high vascularity of the tumour [41], in our study, the experienced surgeon and using recent technology of adrenalectomy helped us immensely decrease the incidence of postoperative bleeding.

PHEO surgery is commonly associated with intraoperative complications and risks [3, 42]. Adhesion to the surrounding tissue with difficult dissection of adhesive perinephric fat is common in patients with a high body mass index, which is common in left-sided PHEO surgery [43]. This may be responsible for intraoperative and postoperative colonic injuries. Logistic regression analysis confirmed that low BMI was associated with risk reduction and intraoperative complications were associated with an increased risk of ER after PHEO surgery. Tumor size has been described as a risk factor for postoperative complications after LA and PHEO surgery [13]. The larger the PHEO, the more frequent the risk of increased vascularity and adhesion to the surrounding structures. Therefore, even with skilled personnel, A PHEO may be associated with increased complications and perioperative mortalities [44]. Our institution used TLA in all scheduled adrenalectomies, regardless of tumour size, similar to previous reports [45, 46]. In the multiple regression analysis, a tumour size of < 5 cm was associated with a risk reduction for ER. Death occurred in two patients (2/49, 4.1%) of readmission in our analysis, and our results suggest the overall safety of perioperative management of patients with PHEO.

## Strengths and limitations

This study has several important limitations. As a retrospective analysis, it was subject to selection bias. A single-country design may limit generalizability to other healthcare systems. Although clinically relevant, outcome definitions lack standardisation (e.g., hypotension duration and ileus criteria). Unmeasured confounders (e.g. outpatient compliance) could influence the results. Nevertheless, this is the first multi-institutional assessment of ER risk factors following PHEO adrenalectomy to identify important associations that warrant prospective validation.

## Conclusions

Postoperative maintained hypotension and ileus are the most common causes of ER. Logistic regression analysis showed that low body mass index, tumour size < 5 cm and low ASA were associated with risk reductions for ER, while malignancy, open approach, and intraoperative complications were associated with increased risk. Preoperative assessment of these risk variables is crucial for surgeons and anaesthetists to determine the likelihood of ER, formulate surgical strategies, minimise admission rates, and enhance postoperative results.

## Data Availability

No datasets were generated or analysed during the current study.
